# Improvement of clinical and radiological outcomes of root repair patients with advanced articular cartilage degeneration and osteoarthritis

**DOI:** 10.1186/s40634-021-00405-3

**Published:** 2021-09-27

**Authors:** Nathan D. M. Hopkins, Steven Lawrie

**Affiliations:** 1 Faculty of Medicine, University of Queensland, Brisbane, QLD Australia; 2 Sunshine Coast Orthopaedic Clinic, Suite 17 Level 2, Kawana Private Hospital, 5 Innovation Parkway, Birtinya, QLD 4575 Australia

## Abstract

**Purpose:**

The main purpose of this study was to investigate clinical and radiological outcomes of medial meniscus posterior root tear (MMPRT) repair in knees with advanced articular cartilage degeneration and osteoarthritis compared to those with minimal degenerative change.

**Methods:**

Thirty-three knees underwent MMPRT repair using an arthroscopic pullout repair tibial tunnel technique. Clinical scores including Lysholm Score, International Knee Documentation Committee (IKDC) Score and Knee injury and Osteoarthritis Outcome (KOOS) Score were collected preoperatively and sequentially at 6 months, 12 months and mean final follow-up of 39.4 months. Kellgren-Lawrence (K-L) osteoarthritis grade, Outerbridge classification of cartilage degeneration and the presence of bone marrow oedema on MRI were also evaluated.

**Results:**

All clinical scores improved at final follow-up for knees with K-L grade ≥ 2 osteoarthritis (*p* < 0.001), with no significant difference compared to K-L 0/1. Patients with Outerbridge class 3/4 cartilage degeneration also reported improvements in clinical scores, albeit lower than those with class 2 degeneration (*p* < 0.05). During recovery, the majority of patients reported clinical improvements by 6 months, and six patients further improved by at least 15 points in IKDC score between 6 and 12 months. Osteoarthritis progressed in 10 of 31 knees (32%), with an overall mean pre-operative K-L grade of 1.6 ± 0.9 compared to 2.0 ± 0.9 at final follow-up (n.s.). No knees progressed to K-L 4 or underwent re-operation. Pre-operative bone marrow oedema was present in 17 knees (52%), all of which had signal localised to the medial tibia or femur. Oedema had resolved in all but 5 knees post-operatively (*p* < 0.01).

**Conclusion:**

Arthroscopic repair of medial meniscus posterior root tears is associated with improved outcomes in knees with advanced cartilage degeneration and osteoarthritis. Meaningful improvements in clinical outcomes can be achieved beyond 6 months, thus success of the operation is best determined at the 12-month mark. Oedema signal significantly improved post-operatively, however a relatively high proportion of knees had K-L progression.

**Level of evidence:**

IV – Case Series.

## Introduction

Integrity of the posterior root of the medial meniscus is essential for normal meniscal function, through maintaining circumferential hoop tension and preventing meniscal extrusion [13, 17]. A complete radial tear of the posterior root disrupts critical circumferential fibres, resulting in a functional total meniscectomy via loss of hoop tension [3]. Without a functional root preventing meniscal extrusion, tibiofemoral contact area decreases and in turn, markedly increases contact pressure [12]. This leads to a pattern of accelerated articular cartilage degeneration [14] and osteoarthritis [2, 17] that is typically seen with this type of injury.

Failing to restore the biomechanical function of the posterior root has important clinical impacts. When managed conservatively, patients with medial meniscus posterior root tears (MMPRT) report poor clinical outcomes [1, 22], are subject to accelerated osteoarthritic progression compared to non-root meniscal tears [6] and undergo high rates of arthroplasty [22]. Partial meniscectomy, which has traditionally been the treatment for medial meniscus root tears, is also associated with poor clinical outcomes [29], and 5-year arthroplasty rates have been reported as high as 35-54% [8, 21].

There has been growing interest and evidence in support of operative treatments that aim to repair posterior root tears by reducing the root to its anatomical position. Biomechanical studies have found that arthroscopic repair of MMPRTs restores contact pressure to normal [12, 28]. It is likely this restoration of the load distributing function of the meniscus that underpins improved clinical outcomes, low reoperation rates at 5–10 year follow-up [1, 7, 9, 18, 23, 24] and slower progression of osteoarthritis [29].

Most root repair studies to date employ strict exclusion criteria; there is a general consensus that root repair is only indicated in patients with mild pre-existing osteoarthritis and low-grade chondral lesions [11]. It is thus unclear whether the benefits that patients with minimal degenerative change report in pain and function extend to those with advanced wear. Furthermore, no studies to date have reported clinical outcomes at multiple post-operative intervals to determine the expected timeline of recovery in pain and function following root repair.

This study therefore aimed to investigate clinical and radiological outcomes of MMPRT repair in knees with advanced articular cartilage degeneration, osteoarthritis and bone marrow oedema compared to those with minimal degenerative change. The primary outcome was patient-reported clinical scores pre-operatively to final follow-up. Secondary outcome measures included radiographic progression of osteoarthritis and resolution of bone marrow oedema post-operatively. The outcomes of this study will help inform orthopaedic surgeons during the shared-decision making process with MMPRT patients with advanced wear, as well as provide insight to both the patient and surgeon about recovery expectations.

## Materials and methods

### Patient selection

**Fig. 1:**
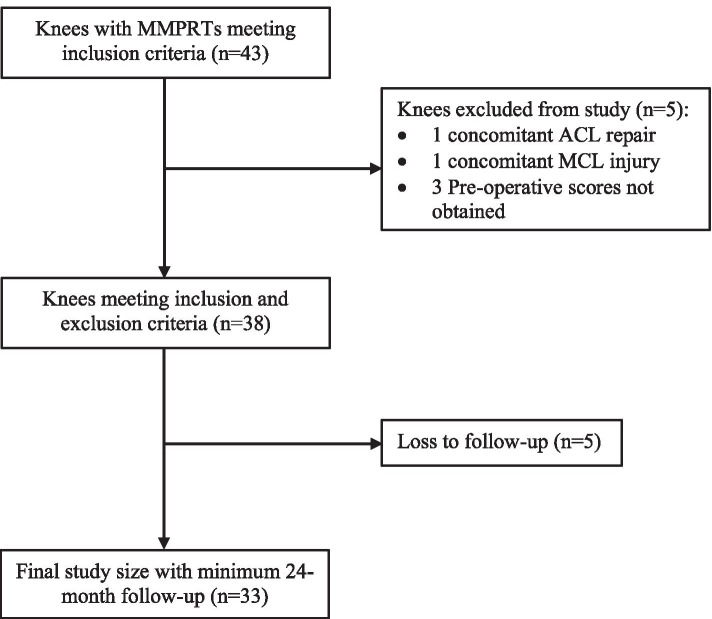
Patient selection process for this study into medial meniscus posterior root tear (MMPRT) repair. Patients were recruited from November 2013 to October 2018

The study protocol was approved by the Uniting*Care* Health’s Human Research Ethics Committee (Reference Number 2016.24.204) and all participants provided informed consent. The inclusion time frame was from November 2013 to October 2018. Patients who were not a current smoker, willing to non-weight bear for the initial 6-week period, had a medial meniscus posterior root tear evident at the time of arthroscopy and had consented to root repair were included. A minimum 24-month follow-up was required. Exclusion criteria of this study included concomitant injury of cruciate ligaments or medial/lateral collateral ligaments, concomitant injury to the contralateral knee, inability to reduce the meniscus root to its anatomical position or meniscal tissue mechanically unsound to hold a suture. Patients were also excluded if pre-operative clinical scores were not obtained. The number of eligible and excluded patients are outlined in Fig. 1.

### Data extraction

Clinical data were collected prospectively as participants completed scores during clinical visits or via post. Scoring was conducted pre-operatively and at 6-month, 12-month and final follow-up intervals. All radiological data were obtained by author S.L. MRI scans were analysed before surgery and at 6 months post-operatively to classify bone marrow oedema. Digital or printed copies of knee radiographs were reviewed to grade the severity of knee osteoarthritis. We were unable to obtain radiographs pre-operatively and at minimum 2 years follow-up for two knees, hence only 31 knees were assessed for progression of osteoarthritis. Intra-operative arthroscopic photos were reviewed by author S.L to grade the extent of cartilage degeneration evident during arthroscopy according to the Outerbridge classification [26]: grade 0, no change; grade 1, softening and swelling of the cartilage; grade 2, fragmentation and fissuring in an area half an inch or less in diameter; grade 3, same as grade 2 but an area more than half an inch in diameter is involved; grade 4, erosion of cartilage down to bone. Outerbridge class 1 or 2 articular cartilage were considered low-grade chondral lesions and class 3 or 4 as high-grade lesions.

### Outcome measures

The Knee injury and Osteoarthritis Outcome Score (KOOS) was used as the primary outcome measure due to its assessment of pain and function across a wide range of daily tasks and impacts of the knee on quality of life. Secondary clinical outcome measures included Lysholm and International Knee Documentation Committee (IKDC) Scores.

Radiological outcomes included radiographic progression of osteoarthritis and change in bone marrow oedema on MRI scans. Severity of knee osteoarthritis evident on knee AP films was graded according to the Kellgren and Lawrence system [19]: grade 0, no radiographic features of osteoarthritis; grade 1, doubtful joint space narrowing (JSN) and possible osteophytic lipping; grade 2, definite osteophytes and possible JSN on anteroposterior weight-bearing radiograph; grade 3: multiple osteophytes, definite JSN, sclerosis and possible bone deformity; grade 4, large osteophytes, marked JSN, severe sclerosis and definite bony deformity. Severity of bone marrow oedema was defined according to intensity and depth of signal as described by Brittberg and Winalski [4].

A failure of the operation was defined as any of the following: (1) not attaining an improvement of at least 15 points or a final score greater than 75 out of 100, (2) reoperation (repeat root repair, high tibial osteotomy or arthroplasty) or (3) worsening bone marrow oedema (increasing intensity or depth of signal). The requirement to improve by a minimum of 15 points was chosen based on test–retest reliability and internal consistency studies that found the minimal clinically important difference in scores to be 10–15 points [20, 31].

### Surgical method

All root repairs were performed by one orthopaedic surgeon at the same hospital using a standard 2-portal arthroscopy. The site of the root attachment was roughened with a chondrotome or curette. A modified ACL tibial drill guide was used to produce a tibial bone tunnel. A tip aimer that has had the tip removed was used, allowing access to the root attachment without damaging the femoral condyle or perforating the medial ligament.

A stab incision was made over the medial tibial plateau and a 2.3 mm beath pin was drilled to the root attachment on the tibial plateau. A Hewson Suture Retriever (tm Smith and Nephew) was then passed up the tibial tunnel into the knee joint. A slotted cannula was passed through the anteromedial portal and through the Suture Retriever loop. Three sutures were then deployed into the root stump using the Curved FAST-FIX 360 system (tm Smith and Nephew). Once all sutures had been deployed, the Suture Retriever was pulled back through the tibial tunnel and with it the long tails. Initially an endobutton with the loop removed was used to fix the suture tails for the first few cases, however due to cost benefits was changed to a 2.3 mm Bioraptor suture anchor for the majority of cases. Average tourniquet time was 15–20 min and root repair was typically performed as a day surgery procedure.

Patients were kept non-weight bearing and placed in a hinge knee brace set at 0–90 degrees for 6 weeks to avoid loading the meniscal root in the initial stage of healing. Deep knee flexion is avoided for six months. Physiotherapy was commenced at 6 weeks and focussed on restoring quadriceps strength and neuromuscular control.

### Statistical analysis

Data were analysed using GraphPad Prism 9 software. Wilcoxin signed-rank tests were used to compare pre-operative and post-operative clinical scores. Mann–Whitney U tests were used to analyse clinical scores at final follow-up with regard to pre-operative Outerbridge class, Kellgren-Lawrence grade, presence of bone marrow oedema, sex and BMI. Fisher exact tests were used for analysing pre- and post-operative presence of bone marrow oedema and K-L grade progression.

## Results

The final study included thirty-three knees from thirty-one patients. Patient demographics of the included patients are summarised in Table 1. All patients at arthroscopy had at least small areas of fragmentation/fissuring of articular cartilage (Outerbridge class 2), with varying degrees of osteoarthritis evident on pre-operative radiographs (K-L grade 0–3). 17 knees (52%) had bone marrow oedema on MRI scans preoperatively; all of which had signal localised to the medial tibia or femur with varying degrees of intensity and depth.

**Table 1:** Patient demographics (*n* = 33)

Characteristic	
Age, yr	56.8 ± 9.7
Female Sex	23 (70)
Follow-up Period, mo	39.4 ± 15.2
Time between injury and operation, wk	12.1 ± 13.1
Body Mass Index (BMI)	30.5 ± 5.3

Demographics are represented as the mean ± standard deviation except for Female Sex expressed as n (%)

*yr* years, *mo* months, *wk* weeks

KOOS, Lysholm and IKDC Scores all significantly improved at a mean final follow-up of 39.4 months (Table 2), regardless of articular cartilage degeneration or osteoarthritic change at time of operation. Patients with high-grade chondral lesions (Outerbridge class 3 or 4) reported significant improvements in clinical scores, albeit to a lesser extent than those with low-grade cartilage degeneration at final follow-up (Table 3). There was no difference in clinical outcomes between knees with K-L grade 2/3 osteoarthritis compared to those with K-L grade 0/1, nor was there a difference in patients who had signs of osteoarthritic progression at final follow-up compared to those with stable radiographs. Of the six patients with K-L grade 3 knees pre-operatively, two patients did not improve. There was also no difference in clinical outcomes at final follow-up based on presence of pre-operative bone marrow oedema (Table 3).

**Table 2:** Comparison of clinical and radiological outcomes preoperatively and at final follow-up

Variable	Preoperative	Postoperative	Difference	*P* Value
Clinical outcomes				
Lysholm Score	41 ± 22	85 ± 17	44 (35–53)	< 0.001^a^
IKDC Score	28.3 ± 18.2	67.3 ± 19.8	39.0 (30.0–48.0)	< 0.001^a^
KOOS Score	40.2 ± 23.6	81.6 ± 18.0	41.4 (31.6–51.1)	< 0.001^a^
Radiological results				
BMO present	17 (52)	5 (15)		< 0.01^b^
Kellgren-Lawrence Grade	3/13/9/6/0	2/6/13/10/0		0.07^b^
0/1/2/3/4				
K-L average	1.6 ± 0.9	2.0 ± 0.9		
Outerbridge classification 0/1/2/3/4	0/0/9/19/5	–		

Clinical outcomes are presented as the mean ± standard deviation. Difference is presented as the mean with 95% confidence interval range. *BMO* Bone Marrow Oedema, expressed as n (%)

*IKDC* International Knee Documentation Committee, *KOOS* Knee Injury and Osteoarthritis Outcome Score

^a^Wilcoxin signed-rank test

^b^Fisher exact test; K-L grades were grouped as < 2 and ≥ 2 to perform test

**Table 3:** Association of pre-operative variables with clinical outcomes at final follow-up

	Lysholm Score	KOOS Score	IKDC Score
**Nominal variables**			
Outerbridge score			
Grade ≤ 2	95	92.2	79.0
Grade > 2	81^b^	77.6^b^	62.9^a^
K-L Grade			
Grade < 2	87	85.8	70.8
Grade ≥ 2	81^n.s^	75.5^n.s^	61.6^n.s^
Bone marrow oedema			
Absent	90	84.5	69.3
Present	80^n.s^	78.8^n.s^	65.5^n.s^
Sex			
Male	82	76.3	61.2
Female	86^n.s^	84.0^n.s^	70.0^n.s^
BMI			
≤ 30 (*n* = 16)	86	82.4	67.4
> 30 (*n* = 17)	84^n.s^	80.9^n.s^	67.2^n.s^

Nominal variable data compares preoperative factors to the mean postoperative clinical score

*IKDC* International Knee Documentation Committee, *KOOS* Knee Injury and Osteoarthritis Outcome Score

Mann–Whitney U tests were used

^a^*p* < 0.05

^b^*p* < 0.01, n.s = not significant

Osteoarthritis by K-L grade progressed in 10 of 31 knees (32%), with an overall mean pre-operative K-L grade of 1.6 ± 0.9 compared to 2.0 ± 0.9 (*p* = n.s.) (Table 2). There were no cases of progression to K-L grade 4. Bone marrow oedema signal significantly improved on follow-up MRI (*p* < 0.01). Five knees (15%) had ongoing oedema postoperatively, four of which had noticeable reduction in intensity and depth of signal and in one case worsened. There were no knees that underwent re-operation.

There were three failures in this study (10%), all due to not attaining improvement of at least 15 points or final score greater than 75. One clinical failure had K-L grade 3 osteoarthritis and Outerbridge class 4 cartilage degeneration, another K-L grade 3 and Outerbridge class 3, and one with K-L grade 2, Outerbridge class 4 and worsening bone marrow oedema post-operatively.

## Discussion

The most important finding of this study is that patients with advanced osteoarthritis, high-grade chondral lesions and pre-operative bone marrow oedema reported improved clinical outcomes after MMPRT repair at final follow-up. This study included knees with articular cartilage erosion down to bone (Outerbridge class 4) and radiographic joint space narrowing with multiple osteophytes (Kellgren-Lawrence grade 3), who are often excluded in root repair studies to date [11]. Patients with Outerbridge class 3/4 articular cartilage improved clinically at final follow-up, albeit to lower levels than Outerbridge class 2 (Table 3). Two other root repair studies have reported clinical outcomes for high-grade chondral lesions. Ahn et al*.* [1] also found that Outerbridge class 3/4 lesions were associated with reduced IKDC and Tegner-Lysholm scores. Moon et al. [24] similarly found that Lysholm and American Knee Society scores were worse for patients with high grades of cartilage degeneration. There was no difference in clinical outcomes for knees with pre-operative K-L grades of 0/1 compared to those with K-L 2/3. One other study by Ahn et al*.* [1] included root repair patients with advanced osteoarthritis at time of operation and similarly found no difference in outcomes in knees with K-L grade 3/4 arthritis. These findings have implications for patients who already have advanced articular cartilage degeneration and osteoarthritis by time of arthroscopy. As seen in this study, it is likely that a significant proportion of knees with MMPRTs will have extensive wear by time of root repair. This may be due to several factors, including the association of root tears with older age [16], the often-trivial mechanism of injury that delays orthopaedic referral and the underlying rapid progression of chondral lesions that can occur within months [14]. As such, a narrowed patient selection approach that only considers root repair in knees with no or mild pre-existing osteoarthritis may preclude many patients from improved outcomes in pain and function.

**Fig. 2:**
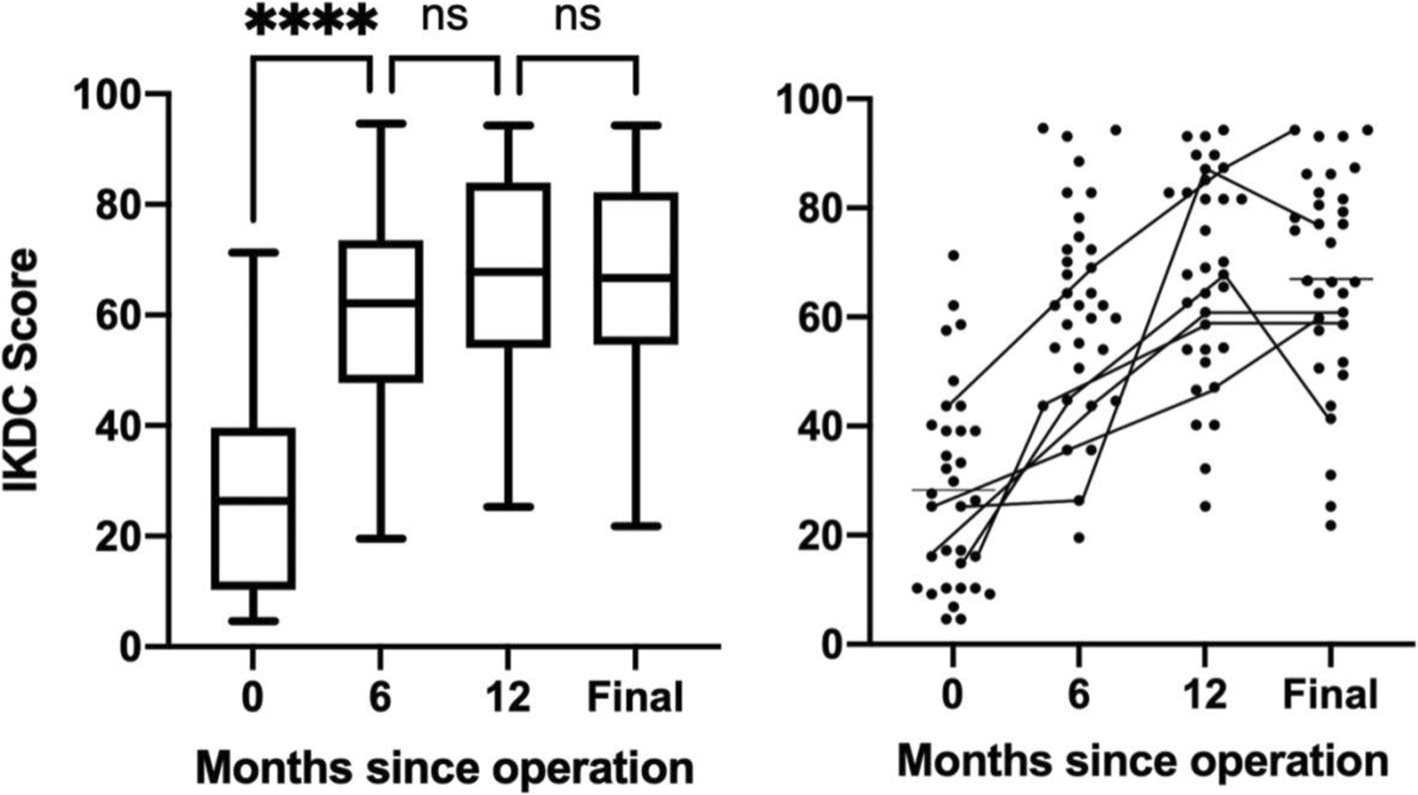
Progression of IKDC scores overtime following root repair (*n* = 33). Scores are presented pre-operatively at 0 months and at post-operative intervals of 6 months, 12 months and mean final follow-up of 39.4 months. Individual trajectories are outlined for the six participants who reported delayed improvements of at least 15 points between 6 and 12 months

Clinical scores improved markedly by 6 months and were maintained overtime (Fig. 2). At an individual level, six patients improved by at least 15 points in IKDC score between 6 and 12 months (Fig. 2), two of which would have met this paper’s clinical failure definition at 6 months. Conversely, no patients who were yet to report improvements at 1 year did so subsequently. This observation highlights that some patients derive benefit beyond 6 months, and that it may be too early to determine the success of the operation at this time point. Rather, patients should be advised about the potential for ongoing improvement and be re-assessed at the 1-year mark.

The average patient in this study was obese with a mean BMI of 30.5. The association of obesity and MMPRTs is becoming increasingly apparent in root tear studies [16, 22, 25, 27]. Biomechanically, it seems likely that obesity is a risk factor given the posterior horn of the medial meniscus is the most fixed compared to other meniscal roots [32] and is therefore more vulnerable to increased load. Despite the association between obesity and this pattern of injury, this study found no significant difference in clinical outcomes for those patients considered normal weight or overweight (BMI < 30) compared to those who were obese. It is not yet clear whether obesity is a poor prognostic factor, with some studies similarly finding no correlation [7, 22], and others reporting worse outcomes in obese patients [5].

Radiographic progression of osteoarthritis was evident in 10 of 31 knees (32%), with a mean K-L grade of 1.6 ± 0.9 pre-operatively and 2.0 ± 0.9 at final follow-up (n.s.). There were no cases of progression to K-L grade 4 or re-operation. A recent meta-analysis by Ro et al*.* compared radiographic outcomes of MMPRTs managed with different operative methods [29]. Authors reported 22.2% of 116 root repair patients developed worse K-L grades at 40.1 months follow-up, compared to 48% managed with partial meniscectomy; an odds ratio of 0.31 (95% CI 0.17–0.54) significantly in favour of meniscal root repair. Although osteoarthritic progression may be slowed by root repair, the proportion of patients who do progress in this study and the wider literature [29] is still relatively high despite restoring hoop tension and the load distributing function of the medial meniscus. This may be due to the extensive cartilage damage that has already occurred in many patients seen at the time of arthroscopic repair. Restoring meniscal root function cannot undo this process; the intention rather is to prevent further rapid articular cartilage degeneration and hence protect the medial compartment from accelerated osteoarthritic progression that is seen with this type of injury.

Preoperative MRIs identified 17 knees (52%) that had bone marrow oedema, all of which had signal localised to the medial tibia and/or femur with varying depths and intensity. The presence of bone marrow oedema largely resolved on MRI at 6-months, where only five knees had oedema still present (*p* < 0.01). Of these, four knees had noticeable reduction in intensity and depth of signal. In one patient, who was a clinical failure as well, bone marrow oedema worsened. Given the localisation of oedema to areas of bone an intact medial meniscus functions to protect, and that patients have been full weight bearing for over 4 months when post-operative MRIs were undertaken, it is implied that the improvement in oedema is due to restoration of meniscal function. Whether this then protects the knee from articular cartilage degeneration is yet to be seen, however this link is plausible given bone marrow oedema is a potent risk factor for structural deterioration of the knee [10] and its progression strongly associated with cartilage degeneration [15, 30].

Limitations of this study include the small cohort size and mid-term clinical follow-up. Clinical outcomes were maintained overtime to a mean follow-up of 39.4 months, however it is possible that clinical deterioration will occur over the longer term, particularly for patients with high-grade chondral lesions and osteoarthritis. This study was also limited by a relatively short-term radiographic follow-up of osteoarthritis. Other MMPRT studies, particularly those investigating non-operative management or partial meniscectomy, follow up patients radiographically for at least 5 years. A similar follow-up period would therefore enable more meaningful insight into osteoarthritic progression overtime in root repair patients compared to traditional management options. Obtaining bilateral knee radiographs pre-operatively and at final follow-up would have also assisted in understanding an individual’s baseline osteoarthritic progression. Another limitation is that this study did not employ multiple raters and relevant interrater tests to collect radiological data, but rather relied on a single rater to determine K-L grades from radiographs and classify intensity and depth of bone marrow oedema on MRI scans.

## Conclusions

Improved clinical outcomes were apparent in patients with high-grade cartilage degeneration, advanced osteoarthritis and bone marrow oedema at time of operation. Meaningful improvements in clinical outcomes were achieved beyond 6 months, thus success of the operation is best determined at the 12-month mark. Oedema signal significantly improved post-operatively, however a relatively high proportion of knees had progression of degenerative changes.
